# Associations Between Isometric Mid-Thigh Pull Peak Force and Functional and Cardiorespiratory Variables in Independent Older Women

**DOI:** 10.3390/jcm15103858

**Published:** 2026-05-17

**Authors:** Jordan Hernandez-Martinez, Izham Cid-Calfucura, Pablo Valdés-Badilla, Pablo Merino-Muñoz, Esteban Aedo-Muñoz, Felipe Montalva-Valenzuela, Pedro Delgado-Floody, Cristian Núñez-Espinosa, Tomás Herrera-Valenzuela

**Affiliations:** 1 Department of Physical Activity Sciences, Universidad de Los Lagos, Osorno 5290000, Chile; 2 Departamento de Educación, Facultad de Humanidades, Universidad de la Serena, La Serena 1700000, Chile; 3 Department of Physical Activity, Sports and Health Sciences, Faculty of Medical Sciences, Universidad de Santiago de Chile (USACH), Santiago 8370003, Chile; 4 Department of Physical Activity Sciences, Faculty of Education Sciences, Universidad Católica del Maule, Talca 3530000, Chile; 5 Sports Coach Career, School of Education, Universidad Viña del Mar, Viña del Mar 2520000, Chile; 6 Escuela de Ciencias del Deporte y Actividad Física, Facultad de Salud, Universidad Santo Tomás, Santiago 8320000, Chile; 7 Núcleo de Investigación en Ciencias de la Motricidad Humana, Universidad Adventista de Chile, Chillán 3780000, Chile; 8 Programa de Engenharia Biomédica, Instituto Alberto Luiz Coimbra de Pós-Graduação e Pesquisa de Engenharia (COPPE), Universidade Federal do Rio de Janeiro, Rio de Janeiro 21941-853, Brazil; 9 Escuela de Entrenador en Actividad Física y Deporte, Facultad de Ciencias Humanas, Universidad Bernardo O’Higgins, Santiago 8370040, Chile; 10 Department of Physical Education, Sport and Recreation, Universidad de La Frontera, Temuco 4811230, Chile; 11 Centro Asistencial Docente e Investigación (CADI-UMAG), Universidad de Magallanes (UMAG), Punta Arenas 6200000, Chile; 12 Escuela de Medicina, Universidad de Magallanes (UMAG), Punta Arenas 6200000, Chile

**Keywords:** muscle strength, cardiorespiratory fitness, functional performance, older adults

## Abstract

**Background/Objectives**: Muscle strength is a key determinant of functional capacity in older adults. However, measures such as handgrip strength may not fully reflect multi-joint force production, and the relevance of the Isometric Mid-Thigh Pull (IMTP) for functional and cardiorespiratory outcomes remains unclear. This study examined the associations between IMTP-derived peak force and functional and submaximal cardiorespiratory variables in independent older women. **Methods**: This cross-sectional study included 21 independent older women (72.6 ± 6.9 years). Maximal isometric strength (IMTP and handgrip), functional performance (TUG, 30-CST, 30-ACT), and submaximal cardiorespiratory variables were assessed. Associations were examined using Pearson’s correlation coefficients with false discovery rate (FDR) correction (q = 5%). **Results**: Absolute IMTP peak force was significantly related to handgrip strength (r = 0.77; q = 0.001) and PVT1 (r = 0.67; q = 0.007). Relative IMTP peak force was related to relative handgrip strength (r = 0.71; q = 0.002), VO_2_VT2 (r = 0.60; q = 0.02), and inversely to COP (r = −0.56; q = 0.03). No significant relationships were observed with TUG, 30-ACT, or most cardiorespiratory variables. **Conclusions**: IMTP-derived peak force was related to selected neuromuscular and submaximal cardiorespiratory variables, but not to functional performance measures. These findings suggest that the IMTP may provide complementary information on neuromuscular status, although further studies are required.

## 1. Introduction

Age-related processes are characterized by a gradual reduction in muscle strength, a decline that has been widely associated with a higher incidence of chronic conditions, reduced functional capacity, and increased mortality risk in older adults [1]. It results from a complex interaction of structural and neuromuscular mechanisms, including the loss of muscle fibers and reductions in cross-sectional area, particularly in type II fibers and alterations in the nervous system, such as motor neuron loss [2,3,4]. Collectively, these changes compromise the ability of older people to perform activities of daily living and appear to disproportionately affect women [5].

Available evidence links muscle weakness to a wide range of adverse outcomes in older adults, including diabetes [6], disability [4,7], sarcopenia [2], cognitive impairment [8], and osteoporosis [9], as well as reduced cardiorespiratory efficiency and increased all-cause mortality [3,4,7]. Given the importance of early detection of muscle strength decline, the use of valid and practical assessment tools is essential in older populations. In this context, maximal strength has emerged as a key biomarker of functional health in aging, given its central role in performing daily tasks and maintaining motor autonomy [4].

Maximal isometric handgrip strength (MIHS) is the most widely used indicator of muscle strength in older people and is frequently considered a clinical biomarker of ageing and overall health status [10,11]. However, it primarily reflects upper-limb muscle strength and does not adequately capture the integrated lower-limb force production required for mobility and functional independence. Despite its widespread use, the reliance on handgrip strength as a global marker of muscle function remains conceptually limited, as it does not adequately represent the multi-joint and lower-limb force production capacity required for locomotion and functional independence. Moreover, the assumption that handgrip strength reflects overall neuromuscular status has been increasingly questioned, particularly in populations where lower-limb function plays a more determinant role in daily performance. In this regard, Martien et al. [12] demonstrated, in a large cohort of older adults, that knee extension strength is a superior predictor of functional performance compared to handgrip strength. Similarly, a recent systematic review reported inconsistent associations between handgrip strength and lower-limb or trunk muscle strength across different populations, highlighting that handgrip should not be used as a standalone proxy of overall muscle strength [13]. Accordingly, several studies have reported that lower-body muscle strength is associated with better functional performance in older people [14,15,16,17,18]. For example, Altubasi [16] identified significant correlations (r = 0.45–0.59; *p* < 0.01) between quadriceps time to peak torque and performance in functional tasks, while Bardstu et al. [19] reported associations between knee extensor strength and functional outcomes such as sit-to-stand performance, gait speed, and timed up-and-go (TUG).

Despite this, most studies have assessed muscle strength at isolated joints, which may not fully reflect the integrated force-production capacity required in daily activities. In this regard, the Isometric Mid-Thigh Pull (IMTP) has been widely used in young and adult populations as a valid and reliable tool to assess maximal multi-joint force production [20,21,22]. Its biomechanical characteristics and low technical demands suggest potential applicability in older people; however, evidence in this population remains limited [23]. The current literature is characterized by several important limitations, including the predominant use of single-joint or upper-limb strength assessments, the scarce application of multi-joint isometric tests such as the IMTP in older populations, and the limited understanding of how integrated strength capacity relates to physiological responses during exercise.

Furthermore, there is a lack of studies integrating cardiorespiratory parameters within this framework [24], despite the physiological plausibility that greater muscle strength may improve movement economy and enhance venous return, thereby contributing to cardiovascular efficiency [25,26]. Although muscle strength has traditionally been linked to neuromuscular performance, emerging evidence suggests it may also influence submaximal exercise responses through mechanisms such as improved neuromuscular efficiency and reduced relative exercise intensity. However, these interactions remain insufficiently explored using integrative approaches combining multi-joint strength assessments and detailed cardiorespiratory variables in older populations.

To our knowledge, no studies have applied the IMTP in independent older women while simultaneously examining its association with both functional and cardiorespiratory parameters. Therefore, this study aimed to examine the associations between IMTP-derived peak force and functional and submaximal cardiorespiratory variables in independent older women. Accordingly, we hypothesized that IMTP-derived peak force would show stronger associations with neuromuscular and lower-body performance measures than with upper-body or multifactorial functional tests and that greater IMTP performance would be associated with selected submaximal cardiorespiratory indicators. Also, given the translational and construct validity of the tests, lower-body assessments should demonstrate a stronger relationship with the IMTP, compared with upper-body tests [27].

## 2. Materials and Methods

### 2.1. Study Design

This study is a secondary cross-sectional analysis of baseline data obtained from a previously published randomized controlled trial [23]. Only variables collected prior to intervention were included in the present analysis in order to examine cross-sectional associations between IMTP peak force and functional and cardiorespiratory variables.

### 2.2. Procedures

All evaluations were performed at the same locations (community and sports centers) within a standardized afternoon time window (2:00–4:00 p.m.), following standardized protocols, on separate days with a 48-h interval between testing sessions, and were administered by the same trained evaluators; no adverse events or musculoskeletal or cardiopulmonary complications were reported during the assessment sessions.

The variables assessed included the following: (i) maximal isometric strength, evaluated using the IMTP and MIHS; (ii) muscle strength, assessed through the 30-s chair stand and 30-s arm curl tests; (iii) functional balance, evaluated using the Timed Up-and-Go test; and (iv) cardiorespiratory fitness indicators, including oxygen uptake (VO_2_VT1, VO_2_VT2), power output, oxygen pulse (VO_2_/HR at VT1 and VT2), respiratory exchange ratio at VT1 (RER VT1), ventilatory efficiency (VE/VCO_2_ slope), oxygen uptake efficiency slope (OUES), and the cardiorespiratory optimal point (COP), along with performance in the 2-min step test [23].

### 2.3. Participants

The present secondary analysis included 21 independent older women drawn from a previously published study [23]. No a priori sample size calculation was performed for this secondary analysis, as the study was based on previously collected data from a randomized controlled trial. Therefore, the final sample size was determined by the availability of participants with complete data for all variables of interest. The sample had a mean age of 72.6 ± 6.9 years, bipedal height of 1.59 ± 0.07 m, and body mass of 65.7 ± 10.2 kg. Participants were recruited from community and sport centers in Chile and were required to meet the following inclusion criteria: (i) women between 60 and 75 years of age; (ii) capacity to comprehend and execute simple verbal instructions; and (iii) functional independence, operationalized as a score ≥43 on the Preventive Medicine Examination for Older Adults (EMPAM) developed by the Chilean Ministry of Health, a standardized national screening instrument assessing cognitive, sensory, and physical–motor domains and classifying individuals according to functional status. Exclusion criteria were defined as (i) presence of disabling conditions; (ii) musculoskeletal disorders or ongoing rehabilitation processes that could compromise physical performance; and (iii) temporary or permanent contraindications to physical activity [23]. The selected age range was aligned with the official Chilean definition of older adulthood (≥60 years) and was intended to ensure sample homogeneity while minimizing variability associated with advanced age, comorbidities, and functional decline.

All participants were informed about the objectives and procedures of the original study and provided written informed consent authorizing the use of their data for scientific purposes, including secondary analyses. The study was conducted in accordance with the principles of the Declaration of Helsinki and was approved by the Institutional Ethics Committee of the University of Santiago de Chile (approval number: N° 392/2024) [23].

### 2.4. Measurements

#### 2.4.1. Anthropometric Variables

Body mass was recorded using a calibrated mechanical scale with a precision of 0.1 kg (Scale-tronix, Chicago, IL, USA), with participants wearing light clothing. Bipedal height was assessed in the standing position using a stadiometer (Seca model 220, SECA, Hamburg, Germany). Body mass index (BMI) was computed as the ratio of body mass to the square of standing height (kg·m^−2^). All anthropometric assessments were performed in accordance with standardized procedures established by the International Society for the Advancement of Kinanthropometry (ISAK) [28].

#### 2.4.2. Cardiorespiratory Fitness

Cardiorespiratory fitness was assessed using a submaximal cardiopulmonary physical exercise test (CPX) conducted with a portable gas analysis system (Cortex Metamax 3B, Leipzig, Germany). Participants performed a graded cycling test using a Technogym Excite™ cycle ergometer, beginning at 30 W, with workload increased in 10 W increments each minute while participants maintained a cadence of 50–60 rpm [24]. Physical exercise was terminated upon attainment of the second ventilatory threshold (VT2), which was identified using ventilatory equivalents and end-tidal oxygen and carbon dioxide pressure criteria according to Wasserman’s graphical methods [29]. From the CPX assessment, the following submaximal cardiorespiratory parameters were derived: oxygen uptake at the first and second ventilatory thresholds (VO_2_VT1 and VO_2_VT2), power output at each threshold, oxygen pulse (VO_2_/HR VT1 and VT2), respiratory exchange ratio at VT1 (RER VT1), ventilatory efficiency (VE/VCO_2_ slope), oxygen uptake efficiency slope (OUES), and the cardiorespiratory optimal point (COP) [24].

Additionally, functional cardiorespiratory capacity was assessed through the 2-min step test [30]. Participants stood upright facing a wall where the midpoint between the patella and the iliac crest was marked. They were instructed to alternately raise each knee above the marked height for two minutes, and the total number of successful steps was recorded.

#### 2.4.3. Timed Up-and-Go (TUG)

Functional balance was assessed using the TUG test, following standardized protocols for older people [30]. Participants were asked to stand up from a seated position in a chair with armrests, walk a distance of three meters, turn, return to the chair, and sit down again. Three trials were performed, and completion time was recorded in seconds. Time was recorded independently by two evaluators using single-beam photocell timing gates (Brower Timing System, Draper, UT, USA). The fastest trial, provided that the protocol was correctly executed, was retained for subsequent analyses.

#### 2.4.4. Isometric Mid-Thigh Pull (IMTP)

The IMTP test was conducted inside a squat rack equipped with an adjustable barbell to ensure individualized positioning. The bar was fixed at mid-thigh height, corresponding to knee and hip joint angles of 125–145° and 155–165°, respectively [31]. Ground reaction forces were recorded using two force platforms (Pasco PS-2142, PASCO^®^ Scientific, Roseville, CA, USA), sampling at 1000 Hz and processed using PASCO Capstone software (version 1.13.4). Participants first completed two submaximal familiarization trials at approximately 70–80% of perceived maximum effort, each lasting 6 s and separated by 3 min of rest. During these trials, positioning and technique were adjusted as necessary, and participants were monitored for discomfort or pain [31]. For maximal trials, participants were instructed to pull “as hard and as fast as possible” following a standardized countdown (“3, 2, 1, go”). The onset of force production was determined using a threshold corresponding to five standard deviations above the baseline signal obtained during a 1 s quiet standing period before the pull [22]. The trial yielding the highest peak force was retained for subsequent analysis. All IMTP trials were performed barefoot, with lifting straps not permitted to minimize variability related to footwear or external assistance [31].

#### 2.4.5. Maximal Isometric Handgrip Strength (MIHS)

MIHS was evaluated with a handheld dynamometer (Jamar^®^ PLUS+, Sammons Preston, Patterson Medical, Warrenville, IL, USA) [32]. Participants were seated with the elbow flexed at 90°, the forearm and wrist in a neutral position, and the shoulder relaxed. The dynamometer handle was adjusted to the first position to optimize grip mechanics. Three maximal efforts were performed with the dominant hand, separated by 120 s of rest, and the highest value was retained.

#### 2.4.6. The 30-s Chair Stand Test (30-CST)

Lower-body muscle strength was also assessed using the 30-s chair stand test [33]. Participants sat on a chair without armrests, with their arms crossed over the chest and both feet positioned flat on the floor. Upon command, they were instructed to stand up fully and sit down as many times as possible within 30 s, ensuring full knee and hip extension at the top of each repetition. Standardized instructions and verbal encouragement were provided. Three attempts were completed with 120 s of rest between trials, and the highest value was retained for subsequent analysis [34].

#### 2.4.7. The 30-s Arm Curl Test (30-ACT)

Upper body muscle endurance was evaluated using the 30-s arm curl test [30]. Participants were seated and performed repeated elbow flexion–extension movements using a 2.27 kg (5 lb) dumbbell held in the dominant hand, maintaining a supinated forearm and neutral wrist position. A standardized demonstration and two practice repetitions were provided before testing to ensure correct technique. Participants were instructed to complete as many repetitions as possible within 30 s while maintaining proper form. Only correctly executed repetitions were counted.

**Figure 1:**
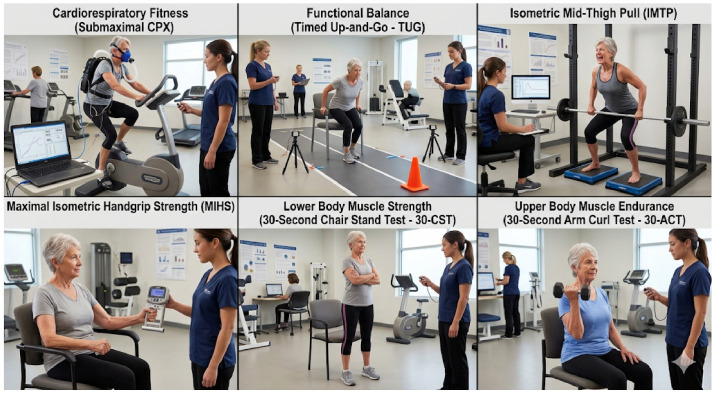
Overview of the assessment procedures, including submaximal CPX, TUG, IMTP, MIHS, 30-CST, and 30-ACT. The figure was generated using artificial intelligence tools and adapted by the authors for illustrative purposes.

An overview of the assessment procedures is provided in Figure 1.

### 2.5. Statistical Analysis

Data are reported as mean ± standard deviation (SD). Assumptions of normality and homogeneity of variance were evaluated using the Shapiro–Wilk and Levene tests, respectively. Additionally, bivariate scatterplots were visually inspected to assess linearity, dispersion patterns, and the presence of potential outliers. No visually apparent extreme outliers were identified. As the assumptions of normality and linearity were satisfied, Pearson’s correlation coefficient (r) was used to examine the associations between IMTP peak force and functional and cardiorespiratory variables. The use of Pearson’s correlation was further supported despite the relatively small sample size, given that the underlying assumptions were met. In addition, relative IMTP peak force (normalized to body mass) was also included in the correlation analyses to account for interindividual differences in body size. The magnitude of the correlations was interpreted as trivial (0.00–0.20), low (0.20–0.40), moderate (0.40–0.60), large (0.60–0.80), and very large (0.80–1.00) [35]. The nominal level of statistical significance was set at α = 0.05. Unadjusted *p*-values were initially reported, and *p*-values were subsequently adjusted for multiple comparisons using the False Discovery Rate (FDR) approach, applying the two-stage step-up procedure of Benjamini, Krieger, and Yekutieli with a desired FDR (q) set at 5% [36]. The FDR correction was applied across all correlation analyses performed between absolute and relative IMTP peak force and the set of functional and cardiorespiratory variables, resulting in a total of 32 comparisons. Given that relative IMTP peak force was included to account for differences in body size, no additional adjustments for body mass or BMI were performed to avoid redundancy between variables. Furthermore, partial correlations controlling for age were not conducted, as the primary aim was to examine direct associations between variables. However, potential age-related influences cannot be ruled out and should be considered when interpreting the findings. Graphical representations display statistical significance based on FDR-adjusted results. All statistical analyses were conducted using GraphPad Prism (version 10.0; GraphPad Software, San Diego, CA, USA).

## 3. Results

Descriptive statistics (mean ± standard deviation) for all anthropometric, TUG, IMTP, MIHS, 30-ACT, 30-CST and cardiorespiratory variables are provided in the Supplementary Materials (Tables S1–S3).

**Figure 2:**
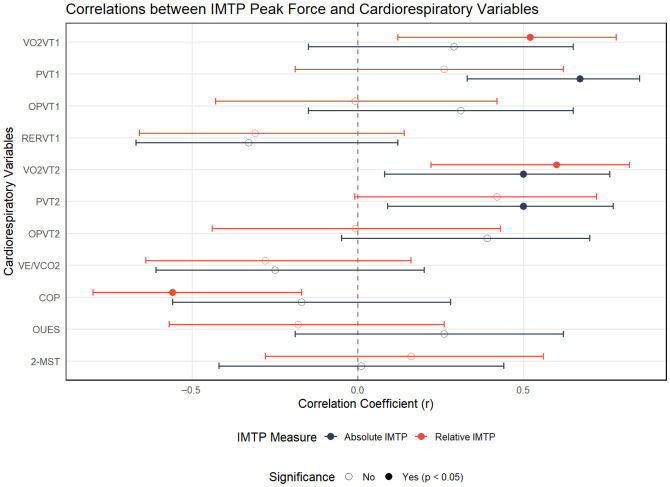
Graphical representation of the correlations between absolute and relative IMTP peak force and cardiorespiratory variables. IMTP: Isometric Mid-Thigh Pull peak force; VO_2_VT1: oxygen uptake at the first ventilatory threshold; PVT1: power at the first ventilatory threshold; OPVT1: oxygen pulse at the first ventilatory threshold; RERVT1: respiratory exchange ratio at the first ventilatory threshold; VO_2_VT2: oxygen uptake at the second ventilatory threshold; PVT2: power at the second ventilatory threshold; OPVT2: oxygen pulse at the second ventilatory threshold; VE/VCO_2_: ventilatory equivalent for carbon dioxide slope; COP: cardiorespiratory optimal point; OUES: oxygen uptake efficiency slope; 2MST: 2-min step test; r: Pearson correlation coefficient; *p*: *p*-value.

**Figure 3:**
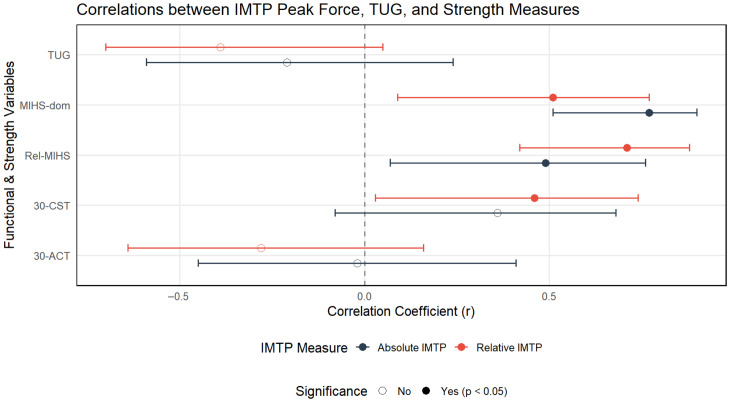
Graphical representation of correlations between IMTP peak force (absolute and relative) and muscle strength and functional performance variables. IMTP: Isometric Mid-Thigh Pull peak force; MIHS-dom: maximal isometric handgrip strength of the dominant hand; Rel-MIHS: relative maximal isometric handgrip strength; 30-CST: 30-s chair stand test; 30-ACT: 30-s arm curl test. TUG: Timed Up-and-Go test.

Table 1 and Figure 2 present the correlations between absolute and relative IMTP peak force and cardiorespiratory variables, whereas Table 2 and Figure 3 report the associations with muscle strength measures and TUG performance. Overall, IMTP peak force was associated with selected cardiorespiratory parameters, particularly PVT1, VO_2_VT2, and COP, as well as with handgrip strength variables, while no significant relationships were observed with functional performance measures.

When controlling for multiple comparisons using the FDR procedure (q = 5%), only a subset of the initially significant correlations remained statistically significant. For absolute IMTP peak force, the positive correlations with MIHS-dom and PVT1 remained significant after adjustment (q = 0.001 and q = 0.007, respectively). For relative IMTP peak force, the positive correlations with Rel-MIHS and VO_2_VT2, as well as the negative correlation with COP, also persisted following FDR correction (q = 0.002, q = 0.02, and q = 0.03, respectively). The remaining correlations did not retain statistical significance after adjustment for multiple comparisons.

**Figure 4:**
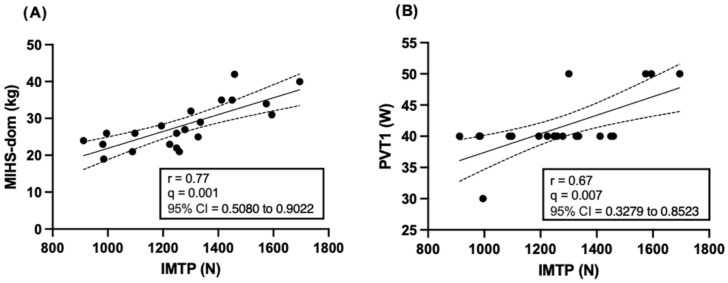
Correlations that remained statistically significant after false discovery rate (FDR) correction between absolute IMTP peak force and (**A**) maximal isometric handgrip strength of the dominant hand (MIHS-dom) and (**B**) power at the first ventilatory threshold (PVT1). Solid lines represent linear regression fits and dashed lines indicate 95% confidence intervals. Correlation coefficients (r), FDR-adjusted q-values, and 95% confidence intervals for r are shown within each panel.

**Figure 5:**
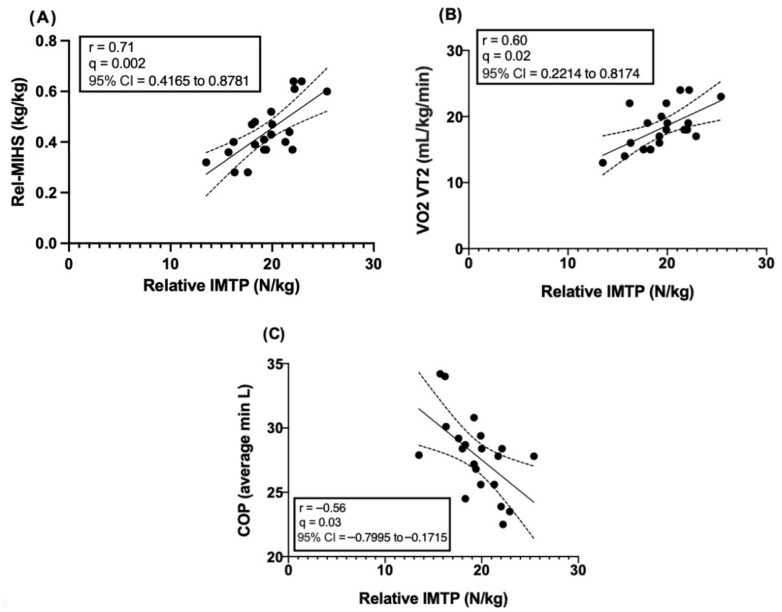
Correlations that remained statistically significant after false discovery rate (FDR) correction between relative IMTP peak force and (**A**) relative maximal isometric handgrip strength (Rel-MIHS), (**B**) oxygen uptake at the second ventilatory threshold (VO_2_VT2), and (**C**) cardiorespiratory optimal point (COP). Solid lines represent linear regression fits and dashed lines indicate 95% confidence intervals. Correlation coefficients (r), FDR-adjusted q-values, and 95% confidence intervals for r are displayed within each panel.

The correlations that remained significant after FDR correction are illustrated in Figure 4 and Figure 5.

## 4. Discussion

This study aimed to examine the relationship between maximal isometric strength, assessed through the IMTP and MIHS, and functional and cardiorespiratory variables in independent older women. In line with our initial hypothesis, the results partially supported the expected correlations, as IMTP-derived peak force was significantly related to neuromuscular and selected cardiorespiratory variables, but not consistently correlated with functional performance outcomes. The main findings were as follows: (i) a large correlation was identified between absolute IMTP peak force and MIHS-dom; (ii) a large correlation was observed between absolute IMTP peak force and power at the first ventilatory threshold (PVT1); (iii) relative IMTP strength was positively correlated with MIHS-rel; (iv) relative IMTP strength showed a moderate positive correlation with oxygen uptake at the second ventilatory threshold (VO_2_VT2); and (v) relative IMTP strength demonstrated a moderate negative correlation with the cardiorespiratory optimal point (COP). Importantly, only a subset of these associations remained statistically significant after correction for multiple comparisons. Therefore, these findings should be interpreted with caution and considered exploratory. Taken together, the results suggest that maximal isometric force production capacity may be related to neuromuscular function and selected aspects of submaximal cardiorespiratory responses in independent older women. However, given the limited sample size and lack of adjustment for potential confounders, these relationships cannot be interpreted as indicative of underlying physiological mechanisms, and further research is required to confirm these observations.

### 4.1. Cardiorespiratory Fitness

A positive correlation was found between absolute IMTP peak force and PVT1 (r = 0.67; q = 0.007), as well as a positive correlation between relative IMTP strength and VO_2_VT2 (r = 0.60; q = 0.02), and a negative correlation between relative IMTP strength and COP (r = −0.56; q = 0.03), after controlling for multiple comparisons using the false discovery rate (FDR) procedure (Q = 5%). Our findings are consistent with those reported by Bardstu et al. [19] in community-dwelling older people receiving home care, who identified significant associations between maximal voluntary isometric contraction (MVIC) of the knee extensors and both preferred walking speed (β = 0.39; *p* < 0.001) and maximal walking speed (β = 0.45; *p* < 0.001). Similarly, Altubasi [16], in a study conducted in older people (61.9% women), reported significant correlations (r = 0.45–0.59; *p* < 0.01) between the time required for the quadriceps to reach peak torque and performance in cardiorespiratory-related tasks, including stair ascent, ramp ascent, and the 4-m walk test.

The literature has consistently shown that aging is associated with a progressive decline in cardiorespiratory fitness, resulting from structural and functional changes that affect multiple physiological systems in an integrated manner [37,38]. At the central level, aging is characterized by a progressive reduction in maximal heart rate and, to a lesser extent, a decline in maximal stroke volume and cardiac output during physical exercise, ultimately limiting the capacity of the cardiovascular system to deliver oxygen to active muscles [38,39]. Concurrently, structural and functional alterations occur within the vascular system, including increased arterial stiffness and a reduced capacity for peripheral vasodilation, which may impair muscle blood flow during physical exercise [38,39]. At the peripheral level, a reduced capacity of skeletal muscle to extract and utilize oxygen has also been described, reflected in a decline in the arteriovenous oxygen difference during physical exercise [39].

Our findings suggest that older women with greater maximal isometric force production capacity, as assessed by the IMTP, may be associated with higher submaximal cardiorespiratory performance, particularly at PVT1 and VO_2_VT2. One possible explanation may relate to neuromuscular efficiency during submaximal exercise [40]. Individuals with greater maximal force production capacity may operate at a lower relative intensity for a given absolute workload, thereby reducing the required motor unit recruitment, particularly of higher-threshold type II fibers [39,40]. This lower neuromuscular demand could contribute to attenuating metabolic perturbations, including lactate accumulation and the associated increase in ventilatory drive, ultimately contributing to a delayed onset of ventilatory thresholds during incremental physical exercise [40]. However, these mechanisms cannot be directly inferred from the present data and should be interpreted with caution. This relationship may be especially relevant in older people, in whom declines in neuromuscular efficiency increase the relative physiological cost of submaximal tasks [3,40,41]. Additionally, greater muscle strength may also be associated with enhanced effectiveness of the skeletal muscle pump, facilitating venous return during exercise [39].

On the other hand, relative IMTP strength showed an inverse correlation with COP, a parameter considered a marker of cardiorespiratory efficiency during submaximal exercise [24]. Lower COP values are typically interpreted as indicators of greater ventilatory efficiency and improved cardiorespiratory performance [26]. The negative correlation observed in the present study suggests a potential association between higher relative muscle strength and more favorable ventilatory responses during physical exercise [3,40]. However, given the exploratory nature of the study, this relationship should be interpreted cautiously and not as evidence of a direct physiological mechanism. Although this relationship has been scarcely explored in the context of maximal isometric strength assessments, it may indicate that muscle function and cardiorespiratory responses are related in independent older women [40].

Importantly, no significant correlations were observed between IMTP peak force (absolute or relative) and variables such as OPVT1, OPVT2, RERVT1, VE/VCO_2_ slope, OUES, or performance in the 2-min step test. In this context, parameters such as VE/VCO_2_ slope and OUES primarily reflect ventilatory efficiency and the integration between pulmonary ventilation, perfusion, and central cardiovascular control during physical exercise and are therefore considered robust markers of global cardiorespiratory function rather than peripheral muscle capacity [24,26]. Similarly, RER at VT1 is associated with substrate utilization dynamics and metabolic regulation during submaximal exercise, processes that are not directly dependent on maximal force production capacity [24,26].

Therefore, the lack of correlations suggests that maximal isometric strength, as assessed by the IMTP, may have a limited association with those components of cardiorespiratory performance predominantly governed by central and metabolic mechanisms [38]. Likewise, the absence of a correlation with the 2-min step test may be explained by its highly multifactorial nature, as performance in this test depends on the interaction between aerobic capacity, coordination, dynamic balance, pacing control, and local muscle endurance [18]. In this context, the specific contribution of maximal strength may be attenuated within a functional framework where multiple physiological systems collectively determine performance outcomes [30].

### 4.2. TUG Perfomance

No significant correlations were observed between absolute IMTP peak force and performance in the TUG test (r = −0.21; *p* = 0.35), nor between relative IMTP strength and TUG performance (r = −0.39; *p* = 0.08). These findings are consistent with those reported by Granacher et al. [42], who found no relationship between maximal isometric strength of the leg extensors and reactive balance in physically active older people. In contrast, Carter et al. [43] reported a significant association (*p* < 0.05) between knee extensor strength and performance in a proactive balance task (figure-of-eight walk), suggesting that the relationship between strength and balance may be task-specific and dependent on the underlying neuromuscular and coordinative demands.

In this context, the lack of significant correlations observed in the present study may be explained by the complex and multifactorial nature of the TUG test, as well as by the specific neuromuscular characteristics assessed by the IMTP. This task involves a sequence of functional actions (standing up, walking, turning, and sitting down) that rely on the integration of multiple systems, including dynamic balance, intersegmental coordination, gait control, and sensorimotor processing, rather than solely on maximal force production [44,45]. Moreover, TUG performance is strongly influenced by temporal and coordinative efficiency, in which factors such as rate of force development, movement strategy, and postural control may play a more prominent role than maximal isometric force production assessed under static conditions [45,46]. Additionally, the IMTP evaluates maximal force in a single isometric effort, which may not adequately reflect the rapid and coordinated force production required during dynamic functional tasks such as the TUG.

Therefore, maximal isometric strength assessed through the IMTP may not represent the primary limiting factor for performance in such functional tasks, particularly in independent older women with relatively preserved functional capacity [46]. Collectively, these findings suggest that maximal force production capacity and functional balance performance may represent partially independent components of physical function in older people. This supports the idea that these capacities should be assessed and trained in a complementary manner when aiming to optimize functional mobility and reduce fall risk [18].

### 4.3. Muscle Strength Performance

A significant correlation was observed between IMTP peak force and MIHS-dom (r = 0.77; q = 0.001), as well as between relative IMTP peak force and Rel-MIHS (r = 0.71; q = 0.002), after controlling for multiple comparisons using the false discovery rate (FDR) procedure (Q = 5%). In contrast, no significant correlations were found between absolute or relative IMTP peak force and performance in the 30-CST or the 30-ACT. These findings are consistent with those reported by Porto et al. [47], who identified a significant positive association between MIHS and overall muscle strength—defined as the sum of maximal torques across ten trunk and dominant lower-limb muscle groups—in community-dwelling older people (r = 0.690; β = 10.07; *p* < 0.001). Similarly, Strandkvist et al. [48] reported that lower-limb muscle strength explained 74.4% (R^2^ = 0.744) of the variance in MIHS in community-dwelling older people.

However, Tatangelo et al. [49], in a narrative review examining the association between MIHS, lower-limb strength, and physical performance in older people, highlighted substantial heterogeneity in the reported correlation coefficients. Specifically, correlations between MIHS and lower-limb strength ranged from trivial (r ≈ 0.00–0.16) to strong (r ≈ 0.60–0.89), depending on the type of contraction assessed, the muscle groups analyzed, and sample characteristics. This variability suggests that MIHS should not be considered a valid surrogate for lower-limb muscle strength.

In this context, the strong correlation observed between IMTP peak force and MIHS in our study may be explained by the similarity in the nature of both assessments, as they reflect maximal force production under isometric conditions [50]. This implies that they share common neuromuscular determinants, including maximal voluntary activation, recruitment of high-threshold motor units, and musculotendinous stiffness [50], which may contribute to the observed consistency between both measures [31]. However, these shared characteristics do not necessarily imply that both tests capture identical aspects of functional performance. To the best of our knowledge, this is the first study to examine the correlation between maximal isometric strength assessed via the IMTP and both functional and cardiorespiratory variables in independent older women.

In contrast, no significant correlations were observed between IMTP-derived variables (absolute or relative) and performance in the 30-CST and 30-ACT. A plausible explanation lies in the multifactorial nature of these tests, albeit with distinct physiological determinants [30]. In the case of the 30-ACT (arm curl test), performance depends primarily on upper-body local muscle endurance and motor control, rather than maximal isometric force production of the lower limbs, which limits its association with IMTP-derived variables [44]. Regarding the 30-CST (chair stand test), although it more directly involves lower-limb muscle, performance is largely influenced by the ability to generate force rapidly and repeatedly—muscle power and movement velocity—rather than maximal isometric strength per se [33]. Additionally, the IMTP assesses maximal force under static conditions, whereas both the 30-CST and 30-ACT involve dynamic, cyclic, and submaximal actions, which may limit the transferability between these measures [31,34]. This discrepancy can also be explained by the principle of specificity, as the IMTP assesses maximal isometric force in a single contraction. In this sense, variables such as muscle power or rate of force development may represent more relevant determinants of performance in these functional tests than maximal strength alone [3,4].

### 4.4. Strengths and Limitations

Our study presented the following limitations: (i) the cross-sectional design precludes causal inference; (ii) the relatively small sample size (*n* = 21) may limit statistical power, increase the risk of Type II error, and affect the stability of the correlation coefficients; (iii) the inclusion of only independent older women restricts the generalizability of the findings to other populations; (iv) the use of bivariate correlations without adjustment for potential confounders (e.g., age, body composition, and physical activity level) should be considered when interpreting the results. However, more complex statistical models were not applied due to the limited sample size, as they may increase the risk of overfitting and yield unstable estimates; (v) although multiple comparisons were conducted, a false discovery rate (FDR) correction was applied to reduce the likelihood of type I error, yet the results should still be interpreted with caution given the exploratory nature of the study; (vi) the study did not assess dynamic neuromuscular variables such as rate of force development (RFD) or muscle power, which are key determinants of functional performance in older people and may be more closely related to tasks such as the TUG or chair stand test; (vii) the IMTP, as an isometric assessment, may have limited ecological validity for dynamic and cyclic functional tasks due to the principle of specificity; and (viii) the use of secondary data from a randomized controlled trial may introduce potential selection bias, which should be considered when interpreting and generalizing the findings.

Conversely, the strengths of our study are the following: (i) to the best of our knowledge, this is the first study to examine the correlation between IMTP-derived maximal isometric strength and both functional and cardiorespiratory variables in independent older women, providing an integrative perspective on neuromuscular function in ageing; (ii) the use of the IMTP allows for the assessment of maximal multi-joint force production under standardized conditions, potentially offering a more representative measure of global neuromuscular capacity than single-joint assessments; (iii) the inclusion of detailed submaximal cardiorespiratory parameters derived from CPX (e.g., ventilatory thresholds and efficiency indices) enhances the physiological relevance of the findings; and (iv) the application of false discovery rate (FDR) correction strengthens the robustness of the results by reducing the risk of type I error.

### 4.5. Practical Applications

The findings of the present study have several practical implications for the assessment and prescription of physical exercise in independent older women:

(i) The IMTP may be considered a useful tool for assessing maximal multi-joint muscle strength in older people, providing complementary information to traditional measures such as MIHS, particularly in relation to lower-limb force production capacity.
(ii) Given the observed correlations between IMTP peak force and selected submaximal cardiorespiratory parameters (e.g., PVT1, VO_2_VT2, and COP), maximal strength assessments may provide complementary information related to submaximal exercise responses, although these findings should be interpreted with caution and not as direct indicators of cardiorespiratory efficiency.
(iii) The lack of correlation between IMTP and functional tests such as the TUG and 30-CST suggests that maximal isometric strength alone may not adequately capture performance in complex functional tasks, which depend on multiple physiological and coordinative factors. Therefore, practitioners are encouraged to include additional assessments targeting muscle power, rate of force development, and balance.
(iv) Physical exercise programs for older people should not rely exclusively on maximal strength training but should also include interventions aimed at improving rapid force production, movement velocity, and functional task performance to better address the multidimensional nature of mobility and fall risk.
(v) From a clinical and applied perspective, the combined assessment of maximal strength, functional performance, and cardiorespiratory fitness may offer a more comprehensive profile of physical capacity, potentially supporting more individualized and targeted intervention strategies.

## 5. Conclusions

In independent older women, IMTP-derived peak force was significantly related to handgrip strength and selected submaximal cardiorespiratory variables, particularly power at VT1, VO_2_ at VT2, and COP. However, these relationships were not consistently maintained after correction for multiple comparisons. These findings suggest that the IMTP may provide useful complementary information on neuromuscular status and selected exercise-related physiological responses. Nevertheless, given the exploratory nature of the study, the small sample size, and the lack of adjustment for potential confounders, further research using larger samples, longitudinal designs, and more robust statistical approaches is required to confirm these findings and better establish their clinical and practical relevance.

## Figures and Tables

**Table 1:** Correlations between absolute and relative Isometric Mid-Thigh Pull peak force and cardiorespiratory variables.

Variables		VO_2_VT1	PVT1	OPVT1	RERVT1	VO_2_VT2	PVT2	OPVT2	VE/VCO_2_	COP	OUES	2-MST
**IMTP**	r	0.29	**0.67**	0.31	−0.33	**0.50**	**0.50**	0.39	−0.25	−0.17	0.26	0.01
	95% CI	−0.15 - 0.65	**0.33** **-** **0.85**	−0.15 - 0.65	−0.67 - 0.12	**0.08** **-** **0.76**	**0.09** **-** **0.77**	−0.05 - 0.70	−0.61 - 0.20	−0.56 - 0.28	−0.19 - 0.62	−0.42 - 0.44
	*p*	0.19	**0.01**	0.17	0.14	**0.02**	**0.02**	0.08	0.26	0.45	0.24	0.94
**Relative IMTP**	r	**0.52**	0.26	−0.009	−0.31	**0.60**	0.42	−0.007	−0.28	**−0.56**	−0.18	0.16
	95% CI	**0.12** **-** **0.78**	−0.19 - 0.62	−0.43 - 0.42	−0.66 - 0.14	**0.22** **-** **0.82**	−0.01 - 0.72	−0.44 - 0.43	−0.64 - 0.16	**−0.80** **-** **−0.17**	−0.57 - 0.26	−0.28 - 0.56
	*p*	**0.01**	0.25	0.96	0.16	**0.004**	0.06	0.97	0.20	**0.008**	0.41	0.47

IMTP: Isometric Mid-Thigh Pull peak force; VO_2_VT1: oxygen uptake at the first ventilatory threshold; PVT1: power at the first ventilatory threshold; OPVT1: oxygen pulse at the first ventilatory threshold; RERVT1: respiratory exchange ratio at the first ventilatory threshold; VO_2_VT2: oxygen uptake at the second ventilatory threshold; PVT2: power at the second ventilatory threshold; OPVT2: oxygen pulse at the second ventilatory threshold; VE/VCO_2_: ventilatory equivalent for carbon dioxide slope; COP: cardiorespiratory optimal point; OUES: oxygen uptake efficiency slope; 2MST: 2-min step test; r: Pearson correlation coefficient; *p*: *p*-value; 95% CI: 95% confidence interval.

**Table 2:** Correlations between absolute and relative Isometric Mid-Thigh Pull peak force and TUG performance, and upper- and lower-body muscle strength measures.

Variables		TUG	MIHS-dom	Rel-MIHS	30-CST	30-ACT
**IMTP**	r	−0.21	**0.77**	**0.49**	0.36	−0.02
	95% CI	−0.59 - 0.24	**0.51** **-** **0.90**	**0.07** **-** **0.76**	−0.08 - 0.68	−0.45 - 0.41
	*p*	0.35	**<0.001**	**0.02**	0.10	0.91
**Relative IMTP**	r	−0.39	**0.51**	**0.71**	**0.46**	−0.28
	95% CI	−0.70 - 0.05	**0.09** **-** **0.77**	**0.42** **-** **0.88**	0.03 - 0.74	−0.64 - 0.16
	*p*	0.08	**0.01**	**0.002**	**0.03**	0.20

IMTP: Isometric Mid-Thigh Pull peak force; MIHS-dom: maximal isometric handgrip strength of the dominant hand; Rel-MIHS: relative maximal isometric handgrip strength; 30-CST: 30-s chair stand test; 30-ACT: 30-s arm curl test. TUG: Timed Up-and-Go test; r: Pearson correlation coefficient; *p*: *p*-value; 95% CI: 95% confidence interval.

## Data Availability

The datasets generated during and/or analyzed during the current research are available from the corresponding author upon reasonable request.

## Supplementary Materials

The following supporting information can be downloaded at: https://www.mdpi.com/article/10.3390/jcm15103858/s1. Table S1. presents baseline cardiorespiratory variables; Table S2. summarizes baseline muscle strength variables; Table S3. reports baseline balance variables for the older adults.
