# Supplementary material for: Associations Between Isometric Mid-Thigh Pull Peak Force and Functional and Cardiorespiratory Variables in Independent Older Women
Source: J Clin Med. 2026 May 17;15(10):3858. doi: 10.3390/jcm15103858 (PMC13207343; doi:10.3390/jcm15103858)
Supplement: Supplementary file 1 [file jcm-15-03858-s001.zip › jcm-4296761-supplementary.pdf]

**Table S1.** Baseline cardiorespiratory variables. Data are presented as mean ± standard deviation with 95% confidence intervals. $VO_2$ = oxygen uptake; HR = heart rate; RER = respiratory exchange ratio; $VE/VCO_2$ = ventilatory equivalent for carbon dioxide; COP = circulatory power; OUES = oxygen uptake efficiency slope; p: significance value.

| Cardiorespiratory variables | Mean ± SD | CI 95% |
|---|---|---|
| $VO_2$ VT1 (mL/kg-min) | 12.8 ± 2.6 | 11.6 – 14.0 |
| Power VT1 (watts) | 41.4 ± 4.7 | 39.2 – 43.6 |
| $VO_2$/HR VT1 (mL/heart beats) | 8.2 ± 1.9 | 7.3 – 9.1 |
| RER VT1 ($VO_2/VCO_2$) | 0.86 ± 0.03 | 0.84 – 0.87 |
| $VO_2$ VT2 (mL/kg-min) | 18.2 ± 3.2 | 16.8 – 19.7 |
| Power VT2 (watts) | 69.5 ± 13.2 | 63.5 – 75.5 |
| $VO_2$/HR VT2 (mL/heart beats) | 9.8 ± 2.3 | 8.7 – 10.9 |
| $VE/VCO_2$ (slope in degrees) | 34.5 ± 5.4 | 32.0 – 36.9 |
| COP (average min L) | 27.8 ± 3.0 | 26.4 – 29.2 |
| OUES (mL) | 1.6 ± 0.07 | 1.58 – |

| | | |
|---|---|---|
| | | 1.65 |
| **2-minute step test** | 101.6 ± 18.1 | 93.3 – 109.9 |

**Table S2.** Baseline muscle strength variables. Data are presented as mean ± standard deviation with 95% confidence intervals. IMTP = isometric mid-thigh pull; N = newton; kg = kilogram; MIHS = maximal isometric handgrip strength; kgf = kilogram-force; p: significance value.

| Muscle strength variables | Mean ± SD | CI 95% |
|---|---|---|
| **IMTP (N)** | 1296 ± 156 | 1184 – 1408 |
| **Relative IMTP (N/kg)** | 19.4 ± 2.9 | 17.32 – 21.58 |
| **MIHS-dominant hand (kgf)** | 28.1 ± 6.3 | 25.1 – 30.9 |
| **Relative MIHS-dominant hand (kgf/kg)** | 0.44 ± 0.10 | 0.39 – 0.49 |
| **30-second chair stand** | 19.4 ± 4.8 | 17.2 – 21.6 |
| **30-second arm curl** | 29.6 ± 5.1 | 27.2 – 31.9 |

**Table S3.** Baseline balance variable. Data are presented as mean ± standard deviation with 95% confidence intervals. TUG = Timed Up-and-Go; s = seconds; p: significance value.

| Balance variable | Mean ± SD | CI 95% |
|---|---|---|
| **TUG (s)** | 5.4 ± 0.72 | 5.1 – 5.8 |
